# Associations of different combinations of moderate-vigorous physical activity and muscle-strengthening activity with mortality among US lung cancer survivors

**DOI:** 10.1186/s12890-024-03108-4

**Published:** 2024-07-05

**Authors:** Weiwei Song, Menglin Zou, Weishuai Zheng, Xingxing Hu, Han Gao, Zhenshun Cheng

**Affiliations:** 1 Department of Respiratory and Critical Care Medicine, Zhongnan Hospital of Wuhan University, Wuhan, China; 2 Fourth Ward of Medical Care Center, Hainan General Hospital, Hainan Affiliated Hospital of Hainan Medical University, Haikou, China; 3 Department of Respiratory and Critical Care Medicine, Hainan General Hospital, Hainan Affiliated Hospital of Hainan Medical University, Haikou, China; 4 Wuhan Research Center for Infectious Diseases and Cancer, Chinese Academy of Medical Sciences, Wuhan, China

**Keywords:** Physical activity, Lung cancer, Outcomes, NHIS

## Abstract

**Background:**

To investigate the associations of different combinations of moderate to vigorous physical activity (MVPA) and muscle strengthening activity (MSA) with all-cause and cancer mortality among lung cancer survivors.

**Methods:**

This nationwide prospective cohort study used data from the US National Health Interview Survey 2009–2018. A total of 785 lung cancer survivors were included in the study. Participants were linked to the National Death Index through December 31, 2019. Self-reported MVPA and MSA frequency data were used to obtain 4 mutually exclusive exposure categories. Multivariate Cox proportional hazard models were applied to explore the association between exposure categories and outcomes.

**Results:**

The mean (standard deviation [SD]) age of the study population was 69.1 (11.3) years and 429 (54.6%) were female. Among them, 641 (81.7%) were White and 102 (13.0%) were Black. The median follow-up time was 3 years (2526 person-years), and 349 (44.5%) all-cause deaths and 232 (29.6%) cancer deaths occurred. Compared to the MVPA < 60 min/week and MSA < 2 sessions/week group, individuals in the MVPA ≥ 60 min/week and MSA < 2 sessions/week group showed hazard ratios (HRs) of 0.50 (95% CI, 0.36–0.69) for all-cause mortality and 0.37 (95% CI, 0.20–0.67) for cancer mortality after the adjustment of covariates. Those in the MVPA ≥ 60 min/week and MSA ≥ 2 sessions/week group exhibited HRs of 0.52 (95% CI, 0.35–0.77) for all-cause mortality and 0.27 (95% CI, 0.12–0.62) for cancer mortality when compared to the MVPA < 60 min/week and MSA < 2 sessions/week group. We also identified distinct non-linear relationships between MVPA and outcomes risk among two MSA frequency subgroups.

**Conclusion:**

This cohort study demonstrated that higher levels of MVPA and MSA combined might be associated with optimal reductions of mortality risk in lung cancer survivors.

## Introduction

Lung cancer is a globally significant disease known for its aggressiveness and high prevalence, accounting for an estimated 2.2 million new cases and 1.8 million deaths in 2020. It holds the unfortunate distinction of being the primary cause of cancer-related mortality among men worldwide and ranks as the second leading cause of cancer death in women, surpassed only by breast cancer [1]. Patients grappling with this condition often endure distressing symptoms, such as dyspnea, cough, fatigue, anxiety, depression, insomnia, and pain, even as treatment efficacy has advanced [2–4]. Non-pharmacological interventions, such as physical activity (PA), have demonstrated their capacity to ameliorate fatigue, enhance quality of life, boost cardiorespiratory fitness, improve pulmonary function, increase muscle mass and strength, and positively influence psychological well-being in individuals with lung cancer [4–6].

Substantial evidence underscores the advantageous impact of moderate to vigorous aerobic physical activity (MVPA) in reducing all-cause and cancer mortality among the general population [7, 8]. These benefits can be further optimized by incorporating regular muscle-strengthening activity (MSA) [9, 10]. With this perspective, the 2018 Physical Activity Guidelines for Americans and the 2020 World Health Organization guidelines on physical activity recommend that adults engage in at least 150 to 300 min of moderate aerobic physical activity (MPA) per week, 75 to 150 min of vigorous aerobic physical activity (VPA) per week, or an equivalent combination of both intensity levels, in addition to MSA on at least two days each week [11].

There is a scarcity of evidence concerning the extent and nature of PA among lung cancer survivors. Thus far, the true potential of PA and exercise in the context of lung cancer remains not fully understood, and there is a notable absence of dedicated exercise guidelines for individuals with lung cancer. Several questions remain unanswered, including the most effective exercise regimen and the real impact of PA on the survival rates of lung cancer patients. The objective of this study was to thoroughly investigate the relationships between various combinations of MVPA and MSA with respect to all-cause and cancer-specific mortality among lung cancer survivors in the United States.

## Method

### Study design and population

This prospective cohort study was a secondary analysis of publicly available and deidentified data from the US National Health Interview Survey (NHIS). The NHIS is an annual nationwide survey conducted by the Centers for Disease Control and Prevention’s National Center for Health Statistics. It aims to provide a representative sample of the civilian, non-institutionalized population in the United States. The National Center for Health Statistics (NCHS) Disclosure Review Board reviewed and approved the NHIS protocol and written informed consent was obtained from all participants [12]. No further institutional review board approval or informed consent was required for the current study. The NHIS design, methodology, and weighting details have been previously published [13]. In summary, the NHIS employed a complex, stratified, multistage sampling design to select households from random clusters. Within these households, a random sample of adults aged 18 years and older were chosen to participate in a comprehensive questionnaire covering aspects of health status, health services, lifestyle risk factors, prevalent diseases, and other health-related matters. Data were collected through personal interviews conducted by trained investigators.

This study included a total of 875 adults with lung cancer history from the 2009 to 2018 NHIS data and their linked records to the National Death Index records through December 31, 2019. We excluded participants who (1) unable to conduct any PA at baseline (*n* = 76); (2) with missing data for PA (*n* = 3); (3) Ineligible for mortality status follow-up (*n* = 11); leaving a total of 785 lung cancer survivor for the analysis of this study. This study adhered to the Strengthening the Reporting of Observational Studies in Epidemiology (STROBE) reporting guidelines [14].

### Diagnosis of cancer

Information about cancer diagnosis and its specific type(s) was obtained through face-to-face interviews, including details about the type of cancer and the age at the time of each diagnosis. Participants were asked, “Ever told by a doctor you had cancer?” Individuals who responded affirmatively were defined as cancer survivors and were further asked, “What kind of cancer was it?” and “How old were you when this cancer was first diagnosed?” [15] The years since cancer diagnosis were calculated as the difference between the participant’s current age and their age at first cancer diagnosis.

### Assessment of PA and MSA

PA information was assessed using the following questions, which were initially validated in the Questionnaire Design Research Laboratory at the National Center for Health Statistics, part of the Centers for Disease Control and Prevention, and subsequently verified through a field pretest [16]: (1) frequency of light-to-moderate activity (MPA): “How frequently do you engage in leisure-time physical activities of light or moderate intensity for a minimum of 10 minutes, activities that lead to only mild perspiration and a slight to moderate rise in breathing or heart rate?”; (2) duration of MPA: “what is the approximate duration of each session when you engage in these light or moderate leisure-time physical activities?”; (3) frequency of vigorous activity (VPA): “How frequently do you participate in vigorous leisure-time physical activities for a minimum of 10 minutes, activities that lead to heavy sweating and significant increases in breathing and heart rate?” and (4) duration of VPA: “About how long do you do these vigorous leisure-time physical activities each time?”. We determined the overall MVPA (in minutes per week) by multiplying the frequency and duration of sessions. The weighted total MVPA was computed by adding the duration (in minutes) of MPA and doubling VPA to account for intensity differences [17, 18].

Muscle-strengthening activity was determined by assessing the frequency of training sessions (times per week) using the following question: “How often do you engage in physical activities specifically designed to enhance your muscle strength, such as weightlifting or calisthenics?” MSA frequency was divided into two groups according to current guidelines: (1) meeting the recommended level (≥ 2 times per week) and (2) below the recommended level (< 2 times per week) [11]. Subsequently, we determined 4 distinct categories that encompassed all possible combinations of MVPA and MSA:1) MVPA < 60 min/week and MSA < 2 sessions/week; 2) MVPA < 60 min/week and MSA ≥ 2 sessions/ week; 3) MVPA ≥ 60 min/week and MSA < 2 sessions/ week; 4) MVPA ≥ 60 min/week and MSA ≥ 2 sessions/ week.

### Ascertainment of all-cause and cancer mortality

The NHIS records of study participants were connected to the National Death Index up to December 31, 2019. The follow-up of this study started with baseline interviews for each participant This data linkage was achieved using a combination of name, social security number, and date of birth, and it exhibited a success rate ranging from approximately 91–98% across survey years. More information about the NHIS data linkage with the National Death Index can be found elsewhere [19]. The mortality outcomes were determined based on the International Statistical Classification of Diseases and Related Health Problems, Tenth Revision (ICD-10) codes, which were recorded as the primary cause of death. In this study, all-cause, and cancer (C00-97) mortality were considered.

### Covariates

The covariates used to adjust the models including the following set of potential self-reported variables: age (years), sex (male or female), race (classified as Black, White, or other[including American Indian or Alaska Native, Asian, Native Hawaiian or Other Pacific Islander, more than one race, or unknown race]), marital status (married/living with partner, divorced/ separated/widowed/never married), smoking status (never, former, or current smoker), alcohol consumption (never, former, or current drinker), history of hypertension, coronary heart disease, stroke, and diabetes, body mass index (BMI; calculated as weight in kilograms divided by height in meters squared), number of diagnosed cancers (excluded lung cancer), functional limitation (yes or no; defined as the participants having difficulty without special equipment in any of the following conditions: (1) walk 1/4 mile; (2) climb 10 steps; (3) stand or sit 2 h; (4) stoop, bend, or kneel; (5) reach over head; (6) grasp small objects; (7) lift/carry 10 pounds; (8) push large objects.).

### Statistical analyses

Categorical variables were expressed as counts and percentages, while continuous variables were presented as mean (standard deviation [SD]). Baseline characteristics were compared using the chi-square test for categorical variables and one-way analysis of variance (ANOVA) for continuous variables. Cox proportional hazard regression models were employed to investigate the relationship between 4 different activity patterns and all-cause and cancer mortality, and the results were reported as hazard ratios (HRs) with corresponding 95% confidence intervals (CIs). The proportional hazards assumption was assessed through the Schoenfeld test. In addition to the crude model, two multivariable models were constructed. In multivariable model 1, adjustments were made for age, sex, race while multivariable model 2 additionally adjusted for smoking, drinking status, diabetes, hypertension, coronary heart disease, and stroke history, BMI, number of diagnosed cancers, and functional limitation. To explore the nonlinear dose-response relationship between moderate-to-vigorous PA (MVPA) and mortality across 2 MSA patterns, restricted cubic spline regression was employed. We also conducted several sensitivity analyses 1). excluding participants older than 75 years of age and those with prevalent heart disease and stroke; 2) to further reduce the possibility of reverse causation bias we excluded mortality occurred during the first 2 years of observation time. All statistical tests were two-sided, and statistical significance was set at *p* < 0.05. All statistical analyses were carried out using R software, version 4.2.0, developed by the R Core Team in Vienna, Austria.

## Result

### Baseline characteristics

Of the 785 lung cancer survivors (mean [SD] age, 69.1 [11.3] years; 429 [54.6%] female) in the study cohort, 102 (13.0%) were Black, 641 (81.7%) were White, and 42 (5.4%) were individuals of Other race (Table 1). Only 28.7% of the cancer survivors were physically active (MVPA ≥ 60 min/wk), while 12.5% reported conducted ≥ 2 MSA per week (Table 1). The detailed baseline characteristics comparison across 4 MVPA and MSA patterns were summarized in Table 1.

**Table 1:** Baseline characteristics of the study population

Characteristic	Overall *N* = 785	MVPA < 60 min/wk and MSA < 2 sessions/wk (*N* = 522)	MVPA < 60 min/wk and MSA ≥ 2 sessions/wk (*N* = 38)	MVPA ≥ 60 min/wk and MSA < 2 sessions/wk (*N* = 165)	MVPA ≥ 60 min/wk and MSA ≥ 2 sessions/wk (*N* = 60)	*p*
**Age, mean (SD), years**	69.1 (11.3)	69.4 (11.0)	69.0 (12.6)	68.9 (11.3)	67.5 (12.5)	0.65
Sex						
Female	429 (54.6)	277 (53.1)	24 (63.2)	95 (57.6)	33 (55.0)	0.53
Male	356 (45.4)	245 (46.9)	14 (36.8)	70 (42.4)	27 (45.0)	
Race						0.14
Black	102 (13.0)	79 (15.1)	5 (13.2)	12 (7.3)	6 (10.0)	
Other	42 (5.4)	25 (4.8)	2 (5.3)	13 (7.9)	2 (3.3)	
White	641 (81.7)	418 (80.1)	31 (81.6)	140 (84.8)	52 (86.7)	
Marital status						0.84
Married or living with a partner	337 (42.9)	221 (42.3)	17 (44.7)	70 (42.4)	29 (48.3)	
Widowed, divorced, or separated	448 (57.1)	301 (57.7)	21 (55.3)	95 (57.6)	31 (51.7)	
Smoking status						< 0.01
Current smoker	153 (19.5)	118 (22.6)	8 (21.1)	24 (14.5)	3 (5.0)	
Former smoker	487 (62.0)	322 (61.7)	24 (63.2)	107 (64.8)	34 (56.7)	
Never smoked	145 (18.5)	82 (15.7)	6 (15.8)	34 (20.6)	23 (38.3)	
Drinking status						< 0.01
Current drinker	378 (48.2)	216 (41.4)	19 (50.0)	102 (61.8)	41 (68.3)	
Former drinker	266 (33.9)	196 (37.5)	14 (36.8)	43 (26.1)	13 (21.7)	
Never drinker	141 (18.0)	110 (21.1)	5 (13.2)	20 (12.1)	6 (10.0)	
Time since lung cancer diagnosis, median [IQR], yeas	3.0 [8.0]	3.0 [8.0]	3.0 [6.8]	4.0 [9.0]	5.0 [8.0]	0.14
Diabetes	179 (22.8)	127 (24.3)	12 (31.6)	30 (18.2)	10 (16.7)	0.13
Hypertension	480 (61.1)	335 (64.2)	24 (63.2)	90 (54.5)	31 (51.7)	0.06
Coronary heart disease	127 (16.2)	91 (17.4)	10 (26.3)	21 (12.7)	5 (8.3)	0.05
Stroke	86 (11.0)	61 (11.7)	9 (23.7)	12 (7.3)	4 (6.7)	0.02
BMI, mean (SD), kg/m^2^	26.6 (6.0)	26.7 (6.3)	28.4 (6.8)	26.0 (5.0)	26.2 (5.0)	0.15
Functional limitation						< 0.01
No	150 (19.1)	72 (13.8)	4 (10.5)	43 (26.1)	31 (51.7)	
Yes	635 (80.9)	450 (86.2)	34 (89.5)	122 (73.9)	29 (48.3)	

Values were displayed as mean (standard deviation) or median [IQR]for continuous variables and count (percentage) for categorical variables

Abbreviations: MVPA, moderate to vigorous aerobic physical activity; MSA, muscle-strengthening activity; SD, standard deviation; IQR, interquartile range; BMI, body mass index

### Survival analysis

**Fig. 1:**
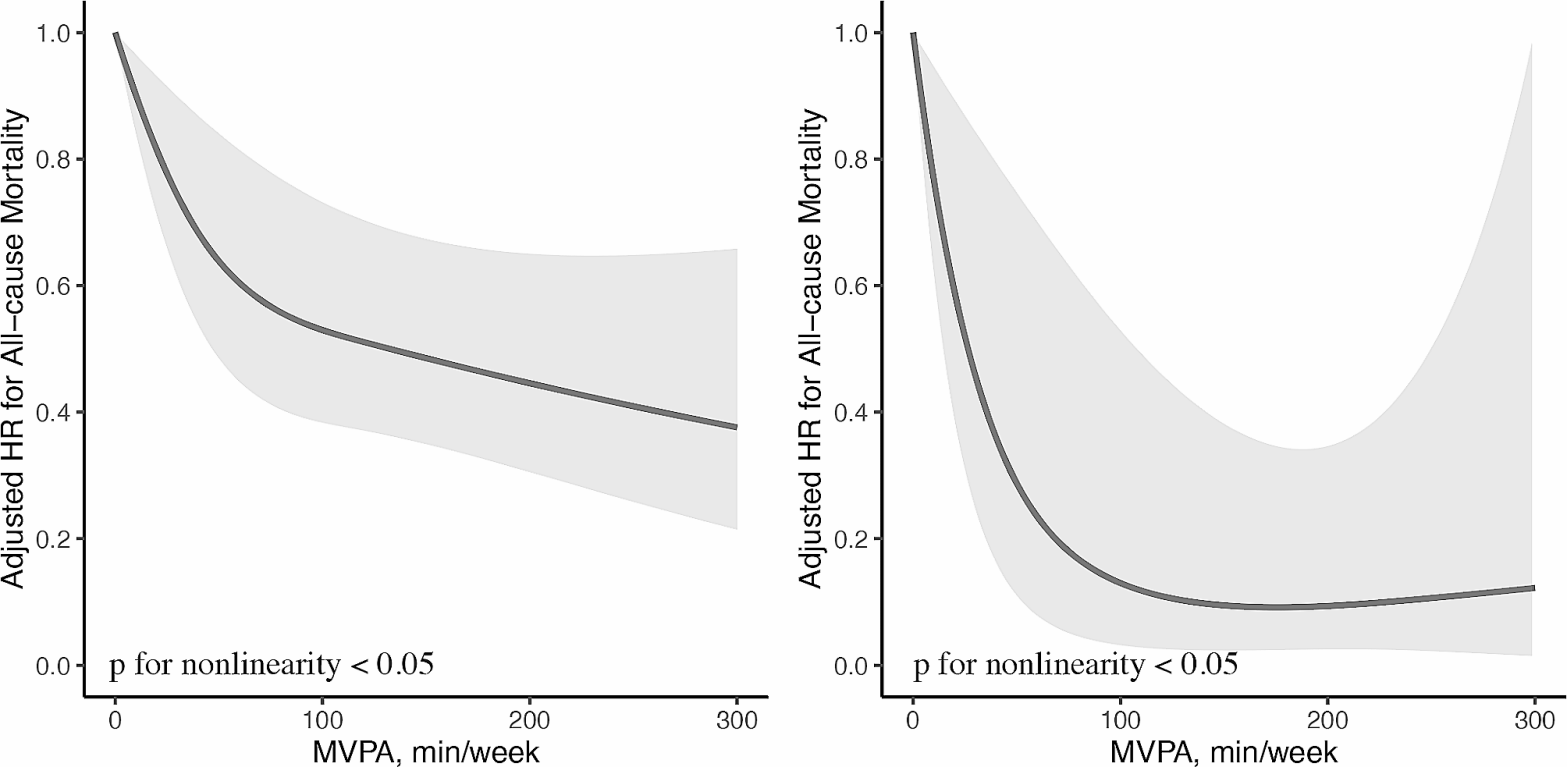
Dose-response relationships between MVPA and all-cause mortality among individuals with MSA < 2 sessions/week (**A**) and MSA ≥ 2 sessions/week (**B**). HRs (solid lines) and 95% confidence intervals (shaded areas) were calculated as per 1 min increase in MVPA and were adjusted for age, sex, race, marital status, smoking, drinking, diabetes, hypertension, coronary heart disease, stroke, BMI, number of diagnosed cancers, and functional limitation. Abbreviations: HR, hazard ratio; MVPA, moderate to vigorous physical activity; MSA, muscle-strengthening activity; BMI, body mass index

**Fig. 2:**
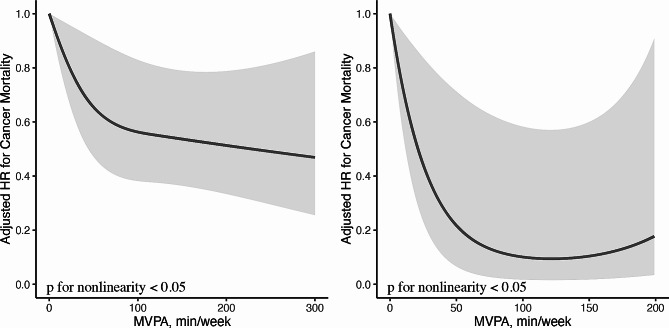
Dose-response relationships between MVPA and cancer mortality among individuals with MSA < 2 sessions/week (**A**) and MSA ≥ 2 sessions/week (**B**). HRs (solid lines) and 95% confidence intervals (shaded areas) were calculated as per 1 min increase in MVPA and were adjusted for age, sex, race, marital status, smoking, drinking, diabetes, hypertension, coronary heart disease, stroke, BMI, number of diagnosed cancers, and functional limitation. Abbreviations: HR, hazard ratio; MVPA, moderate to vigorous physical activity; MSA, muscle-strengthening activity; BMI, body mass index

During the median follow up period of 3 (inter-quartile range, 1–5) years or 2526 person years, 349 (44.5%) all-cause death occurred, including 232 (29.6) cancer mortality. Lung cancer survivors with higher MVPA time and MSA frequency had decreased all-cause and cancer mortality risks (Table 2). After adjusting for potential confounders, there is no significant difference in all-cause and cancer mortality risk between MVPA < 60 min/week and MSA < 2 sessions/week group MVPA < 60 min/week and MSA ≥ 2 sessions/week group (*P* > 0.05; Table 2). However, compared with MVPA < 60 min/week and MSA < 2 sessions/week group, HRs for all-cause and cancer mortality among individuals having MVPA ≥ 60 min/week and MSA < 2 sessions/week group and MVPA ≥ 60 min/week and MSA ≥ 2 sessions/week group were 0.50 (95% CI, 0.36–0.69), 0.37 (95% CI, 0.20–0.67), and 0.52 (95% CI, 0.35–0.77), 0.27 (95% CI, 0.12–0.62), respectively. Among participants with MSA < 2 sessions/week, each 1-h/week increase in MVPA was associated with 21% and 16% reduced risks of death from all-cause and cancer mortality respectively; while in those with MSA ≥ 2 sessions/week, each 1-h/week increase in MVPA was associated with 41% decreased cancer mortality risk (Table 3). In dose-response analysis, non-linear relationships of MVPA and all-cause and cancer mortality were identified in both MSA frequency groups (P for non-linear < 0.05). The curves were steeper and the beneficial effect of MVPA were greater in MSA ≥ 2 sessions/week group (Fig. 1 and Fig. 2). The results were generally robust in sensitivity analysis (Table 4).

**Table 2:** Associations of different combinations of MVPA and MSA with mortality among US lung cancer survivors

	Deaths, *n*	Unadjusted model		Multivariable model 1^†^		Multivariable model 2^‡^	
		HR (95% CI)	*p* value	HR (95% CI)	*p* value	HR (95% CI)	*p* value
**All-cause mortality**							
MVPA < 60 min/wk and MSA < 2 sessions/wk	270 (51.7)	1 (Reference)	/	1 (Reference)	/	1 (Reference)	/
MVPA < 60 min/wk and MSA ≥ 2 sessions/wk	20 (52.6)	0.95 (0.60–1.50)	0.83	1.02 (0.65–1.61)	0.92	1.18 (0.75–1.88)	0.47
MVPA **≥** 60 min/wk and MSA < 2 sessions/wk	47 (28.5)	0.46 (0.33–0.62)	< 0.01	0.47 0.34–0.64)	< 0.01	0.50 (0.36–0.69)	< 0.01
MVPA ≥ 60 min/wk and MSA ≥ 2 sessions/wk	12 (20.0)	0.30 (0.17–0.54)	< 0.01	0.32 (0.18–0.57)	< 0.01	0.37 (0.20–0.67)	< 0.01
**Cancer Mortality**							
MVPA < 60 min/wk and MSA < 2 sessions/wk	180 (34.5)	1 (Reference)	/	1 (Reference)	/	1 (Reference)	/
MVPA < 60 min/wk and MSA ≥ 2 sessions/wk	14 (36.8)	1.00 (0.58–1.73)	0.99	1.04 (0.60–1.79)	0.89	1.18 (0.68–2.05)	0.56
MVPA **≥** 60 min/wk and MSA < 2 sessions/wk	32 (19.4)	0.48 (0.33–0.70)	< 0.01	0.50 (0.34–0.72)	< 0.01	0.52 (0.35–0.77)	< 0.01
MVPA ≥ 60 min/wk and MSA ≥ 2 sessions/wk	6 (10.0)	0.24 (0.11–0.54)	< 0.01	0.25 (0.11–0.56)	< 0.01	0.27 (0.12–0.62)	< 0.01

^†^Adjusted for age, sex, race, and marital status

^‡^Additionally adjusted for smoking, drinking, diabetes, hypertension, coronary heart disease, stroke, BMI, number of diagnosed cancers, and functional limitation

Abbreviation: MVPA, moderate to vigorous physical activity; MSA, muscle-strengthening activity; HR, hazard ratio; CI, confidence interval

**Table 3:** Associations of continues increase of MPA, VPA, and MVPA with Mortality among different MSA frequency group

	Unadjusted model		Multivariable model 1^†^		Multivariable model 2^‡^	
	HR (95% CI)	*p* value	HR (95% CI)	*p* value	HR (95% CI)	*p* value
**All-cause mortality**						
MSA < 2 sessions/week						
MPA (per 1 h increase)	0.63 (0.47–0.85)	< 0.01	0.65 (0.49–0.87)	< 0.01	0.67 (0.51–0.88)	< 0.01
VPA (per 1 h increase)	0.31 (0.15–0.63)	< 0.01	0.34 (0.17–0.69)	< 0.01	0.38 (0.19–0.77)	< 0.01
MVPA (per 1 h increase)	0.76 (0.66–0.87)	< 0.01	0.77 (0.67–0.89)	< 0.01	0.79 (0.69–0.90)	< 0.01
MSA ≥ 2 sessions/week						
MPA (per 1 h increase)	0.65 (0.31–1.34)	< 0.26	0.46 (0.20–1.04)	0.06	0.45 (0.20–0.97)	0.04
VPA (per 1 h increase)	0.02 (0.00-0.25)	< 0.01	0.01 (0.00-0.28)	< 0.01	0.04 (0.00-0.77)	0.03
MVPA (per 1 h increase)	0.63 (0.44–0.92)	0.02	0.53 (0.35–0.79)	< 0.01	0.59 (0.40–0.88)	< 0.01
**Cancer mortality**						
MSA < 2 sessions/week						
MPA (per 1 h increase)	0.74 (0.54–1.01)	0.06	0.76 (0.56–1.03)	0.08	0.77 (0.58–1.02)	0.07
VPA (per 1 h increase)	0.29 (0.12–0.73)	< 0.01	0.29 (0.12–0.74)	< 0.01	0.32 (0.13–0.80)	0.01
MVPA (per 1 h increase)	0.81 (0.70–0.95)	< 0.01	0.83 (0.71–0.96)	0.01	0.84 (0.72–0.97)	0.02
MSA ≥ 2 sessions/week						
MPA (per 1 h increase)	0.62 (0.23–1.67)	0.35	0.50 (0.16–1.54)	0.23	0.85 (0.30–2.37)	0.75
VPA (per 1 h increase)	0.03 (0.00-0.55)	0.02	0.04 (0.00-0.80)	0.04	0.05 (0.00-1.79)	0.10
MVPA (per 1 h increase)	0.63 (0.39–1.01)	0.06	0.57 (0.34–0.97)	0.04	0.79 (0.47–1.33)	0.37

^†^Adjusted for age, sex, race, and marital status

^‡^Additionally adjusted for smoking, drinking, diabetes, hypertension, coronary heart disease, stroke, BMI, number of diagnosed cancers, and functional limitation

Abbreviation: MPA, moderate physical activity; VPA, vigorous physical activity; MVPA, moderate to vigorous physical activity; MSA, muscle-strengthening activity; HR, hazard ratio; CI, confidence interval

**Table 4:** Sensitivity analysis

	Sensitivity Analysis 1^†^		Sensitivity Analysis 2^‡^	
	HR (95% CI)	*p*value	HR (95% CI)	*p*value
**All-cause mortality**				
MVPA < 60 min/wk and MSA < 2 sessions/wk	1 (Reference)	/	1 (Reference)	/
MVPA < 60 min/wk and MSA ≥ 2 sessions/wk	1.05 (0.58–1.89)	0.88	1.01 (0.66–1.63)	0.92
MVPA **≥** 60 min/wk and MSA < 2 sessions/wk	0.46 (0.30–0.70)	< 0.01	0.62 (0.42–0.86)	0.02
MVPA ≥ 60 min/wk and MSA ≥ 2 sessions/wk	0.31 (0.14–0.72)	< 0.01	0.53 (0.41–0.68)	< 0.01
**Cancer Mortality**				
MVPA < 60 min/wk and MSA < 2 sessions/wk	1 (Reference)	/	1 (Reference)	/
MVPA < 60 min/wk and MSA ≥ 2 sessions/wk	0.78 (0.36–1.68)	0.53	1.04 (0.61–1.78)	0.88
MVPA **≥** 60 min/wk and MSA < 2 sessions/wk	0.51 (0.32–0.81)	< 0.01	0.73 (0.41-1.00)	0.05
MVPA ≥ 60 min/wk and MSA ≥ 2 sessions/wk	0.20 (0.06–0.66)	< 0.01	0.32 (0.12–0.86)	< 0.01

^†^ Excluding participants aged 75 years or older

^‡^ Excluding participants died within 2 years follow-up period

Abbreviation: MVPA, moderate to vigorous physical activity; MSA, muscle-strengthening activity; HR, hazard ratio; CI, confidence interval

## Discussion

Using a US national-wide prospective cohort of lung cancer survivors, this prospective study provides new insights into the association among different combinations of MVPA and MSA with long-term all-cause and cancer mortality. Over a follow-up period of up to 9 years, the study unveiled a compelling link between PA and mortality among lung cancer survivors. Specifically, lung cancer survivors who engaged in higher MVPA and more frequent MSA simultaneously exhibited a substantial decrease in all-cause and cancer mortality risk. Notably, the beneficial impact of MSA was most pronounced when accompanied by higher MVPA. Intriguingly, the study identified non-linear relationships between MVPA and outcomes in different MSA frequency groups. The beneficial effect of MVPA was more pronounced in the MSA ≥ 2 sessions/week group, underscoring the importance of varying activity patterns and their potential impact on survival outcomes. However, according to our results, the MSA alone does not necessarily have a significant impact on mortality.

PA is a well-established health promotor both in the general population and cancer survivors [20–24]. Cao et al. reported that among US cancer survivors, the coexistence of extended periods of sitting and a lack of PA was widely prevalent and linked to the greatest risks of all-cause and cancer-related mortality [24]. A meta-analysis found 40–50% relative risk reductions for mortality for breast, colon and prostate cancers with high versus low levels of PA [24]. PA as a non-pharmacological intervention seems to have a large potential to reduce mortality in lung cancer patients. However, considering the poorer health status and physical limitations compared to the general population, the participation of PA among cancer survivors is relatively low. it remains a significant public health challenge to encourage PA participation among lung cancer patients.

The biological mechanisms of how PA improves the survival of lung cancer patients are not fully understood. PA may counteract certain cancer cell characteristics, known as “hallmarks of cancer,” and mitigate chemotherapy-related adverse effects [2]. Cancer cells often evade cell death and apoptosis, regulated by the p53 tumor suppressor protein [25]. PA may promote p53-induced apoptosis, as seen in mouse models of lung adenocarcinoma [26]. The phosphoinositide 3-kinase–AKT pathway and the RAS‐MAP kinase cascade enhance cell proliferation and survival, contributing to chemotherapy resistance in lung cancer cells [27, 28]. PA has shown potential to reduce cell proliferation and survival, likely by downregulating these pathways [29]. Immunomodulation is another possible mechanism, with PA and exercise increasing proinflammatory cytokines and natural killer (NK) cell infiltration in the tumor microenvironment [30, 31]. Studies have demonstrated that exercise can reduce tumor volume, upregulate proinflammatory cytokines, and enhance NK and T-cell activity [32, 33].

Although there was extensive research quantified the relationship between PA time and outcomes, few studies focused on the PA patterns or combinations of different types of activities [16, 18]. To our knowledge, this is the first study to investigate the association of PA and MSA patterns among lung cancer survivors. These findings showed that the optimal combination of MVPA and MSA associated with lower mortality risk of all-cause, and cancer requires a contribution of both 2 different types of PA. The findings contribute valuable insights into the role of PA in the survival of lung cancer survivors, shedding light on the optimal activity patterns associated with reduced mortality risk.

Our findings hold significant implications for the care and well-being of lung cancer survivors. Emerging evidence indicates the safety of PA for lung cancer patients, whether post-surgery or during and after medical treatments [2]. Diverse programs encompassing activities like tai chi, aerobic and strength exercises, walking, balance exercises, and breathing techniques have been explored [33–36]. The most common exercise frequency was two to three times a week, with sessions lasting from 5 to 120 min. Across these studies, patients generally tolerated all reported training intensities, including light, moderate, and vigorous. However, it’s important to note that many lung cancer patients remain insufficiently active or sedentary, leading to low adherence and high dropout rates in various exercise programs [37, 36]. Reasons for dropout often include cancer-related side effects, with the primary contributor being a lack of interest and motivation. Our results suggest that a multifaceted approach to PA, combining both MVPA and MSA, may be optimal for reducing mortality risk. This insight can guide healthcare professionals in developing tailored exercise recommendations for lung cancer survivors, emphasizing the synergistic effects of different types of PA. Furthermore, the study underscores the importance of promoting PA as an integral part of survivorship care. Encouraging lung cancer survivors to engage in regular MVPA and MSA may not only enhance their overall quality of life but also contribute to longer-term survival.

### Strengths and limitations

This study benefits from several strengths, including its use of a nationally representative sample, a lengthy follow-up period, and the consideration of a wide array of potential covariates. The sensitivity analysis supported the robustness of the findings, even when accounting for factors like age and prevalent diseases. However, some limitations should be acknowledged. Firstly, the study relied on self-reported data, which may introduce recall bias. Secondly, the presence and diagnosis time of lung cancer is based on self-reported questionnaires which were inevitable to recall biases. Thirdly, the study focused solely on lung cancer survivors and may not be generalizable to survivors of other cancer types. It is also important to recognize that while the study identified associations, causation cannot be definitively established in observational research. Finally, the cancer stage and treatment information were unavailable in the NHIS dataset. The lack of this information could potentially confound the observed associations between physical activity patterns and outcomes. The heterogeneity in cancer stage and treatment regimens among lung cancer survivors may lead to varying levels of physical activity engagement and subsequent outcomes. The absence of this granularity in our data prevents us from conducting subgroup analyses or adjusting for these important confounders adequately.

In conclusion, this prospective cohort study of lung cancer survivors provides valuable insights into the intricate relationship between PA and mortality. The results suggest that a combination of moderate-to-vigorous PA and muscle-strengthening activity is associated with reduced all-cause and cancer mortality risk. The study’s findings encourage healthcare professionals to emphasize the importance of a multifaceted approach to PA when developing survivorship care plans for lung cancer survivors. Further research is warranted to explore these relationships in more diverse cancer survivor populations and to delve deeper into the mechanisms underlying these associations.

## Data Availability

The datasets generated during and analyzed during the current study are publicly available on the NHIS website (https://www.cdc.gov/nchs/nhis/data-questionnaires-documentation.htm).
